# A phenomenological insight into what final year undergraduate student nurses perceive is the role of the Registered Nurse and who they learn this from

**DOI:** 10.1177/17449871221139112

**Published:** 2022-12-14

**Authors:** Nicola Morrell-Scott

**Affiliations:** Programme Manager Pre-registration Nursing/Associate Dean Education, School of Nursing and Allied Health, Liverpool John Moores University, Liverpool, UK

**Keywords:** holistic care, learning, nurse’s role, partnership working, pre-registration nurse, professional practice

## Abstract

**Background:**

This paper considers the perceptions of 18 final year student nurses around their perception of the role of the nurse.

**Methods:**

A qualitative phenomenological research study was undertaken, with final year student nurses as the data source, undertaking semi-structured interviews from a United Kingdom Higher Education Institution. Data analysis was undertaken by using interpretative phenomenological analysis.

**Results:**

Findings indicate that student nurses had little insight as to the role of the nurse when commencing the programme. Being on the programme has led them to understand there are many aspects of a nurse’s role including, surprisingly for them, much responsibility. It was also found that a lot of the learning that takes place comes from working with healthcare assistants, and due to this, the student nurses perceive that the role of the modern nurse is task orientated and there is little holistic care.

**Conclusions:**

The future practice of this group of registrants may be affected depending on how they move forward with their role, alongside the holistic role of the nurse being detracted from. While phenomenology does not account for generalisations but does seek to illuminate this phenomenon; however if this view were to be shared globally, then the caring aspect of nursing may well be in danger of being lost. If this assumption of the role of the nurse is shared globally, then there are inevitably implications for the nursing profession, and more importantly patient care may be affected.

## Introduction

The role of the nurse is vast and wide in its range. The International Council of Nurses (ICN) defines nursing as:

Nursing encompasses autonomous and collaborative care of individuals of all ages, families, groups and communities, sick or well and in all settings. It includes the promotion of health, the prevention of illness, and the care of ill, disabled and dying people. Advocacy, promotion of a safe environment, research, participation in shaping health policy and in patient and health systems management, and education are also key nursing roles. (ICN, 2016)

The health needs of any nation are said to be critically dependent on the relevant and appropriate education of its nurses (Turale, 2011). While it is essential that new registrants are adequately prepared for the technical and social complexities of the healthcare system, fundamentally nurses are required to provide holistic care to patients. It is difficult to define ‘care’, as each person has differing criteria as to what ‘care’ should be. Difficulties of defining care have been acknowledged, and this is summarised by the seminal work of Smith (1992), ‘Care as a concept is complex. It is referred to on several different levels. Nursing leaders exhort nurses to care, but their definitions are limited because they fail to consider the emotional complexities of caring’ (Smith, 1992: 135). Providing care for patients is extremely important, as it is the fundamental component of what a good nurse is, which then forms the foundations of any technical ability and physical tasks (Gray and Smith, 2009; Smith, 1992). This is fundamentally what the role of the nurse is, to provide care for patients and their families.

This research investigates the perceptions of student nurses, to gain an insight into what they perceive to be the role of the modern nurse is, as they near their own entry to the profession. In much of the literature, there is debate about what the modern nurse does, and is, with debates and definitions going on since the 1960s, yet Scott et al.’s (2014) summary was useful, which described how nurses need to be able to undertake the technical tasks to provide patient care but also need to be intelligent and caring to allow patients to feel properly care for, and they highlight that patients will often have a better level of satisfaction from nurses who they perceive are more caring. Nurses are no longer a physician’s assistant but are a vital and expensive contributor to the healthcare system.

### Background

Pre-registration nurse education within the United Kingdom (UK) currently consists of 50% of time taking place within the university setting and 50% taking place within the clinical environment (Nursing and Midwifery Council (NMC), 2018). Following students completing the programme of study where they have passed theory and practice elements, they are eligible to apply for registration with the NMC, as the students would be fit for practice and for award. This split is thought to produce well-rounded Registered Nurses (RNs) at the end of the programme. Student nurses must complete a minimum of 2300 hours of both theory and practice time, totalling 4600 hours, in a minimum of 3 years and achieve the agreed NMC criteria to gain their professional registration (NMC, 2018). The NMC, the regulatory body for nurses and midwives within the United Kingdom, set the new standards for nursing education, which university providers abide by were reviewed in 2018 following consultation.

This includes points that nurse education should keep core values such as: how the delivery of patient centred care should be at the centre of all pre-registration nursing, safety and dignity should be embedded as a top priority and nurses should be prepared to work within a variety of settings for now and the future. Holistic care was at the centre of the Willis’ (2015) review and is central to the National Health Service (NHS) Constitution (Department of Health (DH), 2015). Often the holistic role of the nurse has been overtaken in view of undertaking day-to-day tasks that are required for the modern healthcare system (King and Gates, 2006; Olive, 2003). Yet it is likely that while students may be undertaking care holistically, they are separating the tasks as part of their proficiencies, and not seeing how this all fits together to form the holistic care of the patient.

Nurses of the 21st century need to be able to have the relevant knowledge and skills to be able to care for a diverse array of patients safely and competently (Nielsen et al., 2013), again supporting the rationale for this research to assist with curriculum enhancement through informing nursing educators to illuminate early on in their programmes what nursing is as a role. There are a variety of changing complexities and challenges when caring for patients; these include inadequate staffing, budget constraints and a lack of practice placement experiences (Ironside and McNelis, 2010; Nielsen et al., 2013; Spence et al., 2012) as issues which will negatively impact on student nurses’ learning.

It was emphasised by the DH in 2010 that the pre-registration nurse education standards should reflect the changes of the healthcare context and should allow for all new nurses qualifying to have the skills to be able to work with competence, across differing healthcare settings. The 21st century provides challenges in terms of educating undergraduate nurses (McNamara, 2014). Caring behaviour has been suggested to lessen as students move towards the end of the programme (Curtis et al., 2012; Mackintosh, 2006; Murphy et al., 2009). However, these are not new points, as this was noted as long ago as Smith (1992) who described how student nurses commence programmes with enthusiasm, but by the end of the programme, they display cynicism and disillusionment. Along with these traits, students also become less focused on the patient and more focused on the illness, along with not demonstrating an emotional side to caring. It has been suggested that care diminishes for student nurses towards the end of their programme (Maben et al., 2007). This then leads to students becoming dissatisfied and disappointed about the profession that they are entering into because the realities of practice pressure hit them (Maben et al., 2007).

## Methodology

### Design

The aim was to undertake a research approach that would examine participants’ lived experiences, to allow conclusions to be made when answering the research questions, hence the employment of a phenomenological approach. The theoretical lens for this research was constructivism, correlating with the use of phenomenology, due to both having an emphasis on how the meaning and thoughts are understood. The research was qualitative in nature and utilises a phenomenological approach.

Phenomenology aims to discover and develop understanding of experiences as identified by those living the experience (Polit and Beck, 2010; Rebar et al., 2011). The lived experiences of the participants, both positive and negative, are important (Liu et al., 2010), and gaining an insight as to student nurses’ perspectives of what the role of the nurse is is important to ascertain while nursing is changing at a rapid rate.

### Participants

A population of 98 adult field final year pre-registration nursing students from one UK Higher Education Institution (HEI) were the total potential population. From this, 18 adult field students self-selected to be a part of the research from this group. None of the sample group withdrew from the study once they had agreed to be in it. This sampling strategy was utilised as it is deemed as being reflective of the larger population (Gray, 2014). Purposive samples have been used in many studies relating to nursing and nurse education from large cohorts (Laurencelle et al., 2016; Watts and Davies, 2014), providing assurance that this was an appropriate method of sampling strategy.

#### Participants were chosen who met the inclusion and exclusion criteria:

*Inclusion criteria* – any third-year pre-registration student nurse.

*Exclusion criteria* – any first- or second-year pre-registration student nurse. Justification for using this group of participants was due to both their knowledge and experience of being a pre-registration student nurse. Participants were currently ‘living’ both the curriculum and the hospital experience through placements. Therefore, they had the knowledge and experience of what they felt was needed to practice, and what was being taught. Final year student nurses as a group have been utilised in previous research to examine experiences and perspectives (Watts and Davies, 2014). Other research studies have found it beneficial to utilise final year nursing students as participants (Felstead and Springett, 2016; Watts and Davies, 2014).

#### Participant name

Collecting the demographic data was important, so that after analysis had been completed in line with the phenomenological paradigm used, a richness of the student’s experience could be generated.

### Data collection

To achieve the aims of the research and answer the research questions appropriately, the method of data collection chosen was semi-structured interviews. The key work by Oppenheim (1996) suggests how questions need to be composed and improved upon to yield the answers that are required. In remaining true to the tenets of the qualitative paradigm, a pilot interview schedule was not undertaken. Guidance in this process is established in qualitative research literature (Oppenheim, 1996). The depth and detail required to answer the research questions meant that semi-structured interviews were the most appropriate data collection method for this research given that this could produce the level of the detail and depth to answer the research questions (Gray, 2014), linking with the qualitative phenomenological approach. The audio-recorded, semi-structured interviews took place on a one-to-one basis with the participant and the researcher and lasted on average around an hour per participant. Other nursing research studies (Clucas and Chapman, 2014; Felstead and Springett, 2016; Valizadeh et al., 2016) have used this approach with similar durations of interviews, which provided the researcher assurance that the interview timings were appropriate. An interview schedule was followed as a guide, to allow some standard questions to be asked and keep focus.

### Ethical considerations

Ethical approval was granted through the university in which the study took place, approval reference no: RS2014/95. In compliance with British Educational Research Association (2011) guidelines, the NMC (2018) Professional Code and the Royal College of Nursing (2011) research guidelines’ professionalism was maintained. Participants were treated with fairness, dignity and sensitivity. All participants were given pseudonyms to ensure confidentiality and anonymity are adhered to.

### Trustworthiness

Research findings should be trustworthy, in respect of the procedures used to generate the themes (Graneheim and Lundman, 2004). Trustworthiness is essential to capture the phenomenon of interest within a study (Lauckner et al., 2012). Within the findings section, excerpt quotes are used with pseudonym names to allow the reader to gain an understanding of the themes. Trustworthiness is guaranteed as processes were undertaken to ensure that the data analysis process was conducted with rigour.

### Data analysis

The method of data analysis adopted for this research was interpretive phenomenological analysis (IPA). IPA aims to explore in depth and detail the lived and personal experiences of participants (Lyons and Coyle, 2007). As this aligns with the phenomenological aspects of this study, it makes it the most appropriate method of data analysis for the study. Considering what phenomenology is, it is literally the study of phenomena, whatever the phenomena may be. IPA aims to explore how sense is made of the participants’ social and personal world, including their experiences (Smith and Osborn, 2008). Themes derived from data analysis were as follows:

Prior assumptions of the role of the nurse
Nursing socialisation
View of the role of the nurse for now and the future

## Results

The results are discussed below with pseudonyms for participant names.

### Prior assumptions of the role of the nurse

An important aspect of the findings was the insight into the roles of the RN that student nurses had before commencing the course. It appears that the distinction between the role of the student nurse and RN is something that many final year participants had little insight into when beginning the programme. Participants throughout the sample described how they did not have understanding into what the role of the nurse was, when commencing the programme. Interestingly there was also no difference between those who had healthcare experience and those who did not, in terms of their assumption of the role of the nurse. Participants were not asked what experience they had prior to entering the programme unless they volunteered this information; therefore, it was difficult to understand how much time they spent with RNs to understand the role of the nurse. One participant, Maureen, who did have previous healthcare experience, stated, ‘nobody told me that there would be so many things involved in being a nurse and what the role consisted of’ (Maureen). Considering Maureen did have healthcare experience, it is surprising her lack of insight as to the role of the nurse. This is interesting as to what participants believed that the role of the nurse was, when applying for the programme, and what drew them to the profession in the first place. Deborah, who had no healthcare experience, highlights not only her lack of knowledge of the role of the nurse but also the programme, ‘I never even realised that I would have to work shifts . . . my god back then I really was clueless’ (Deborah).

The lack of understanding may question the recruitment and selection process in terms of explaining the awareness of the role, and the programme, that participants had, but it is also important that as candidates have this explanation that they also have the information provided at the right time and in the right way to ensure understanding. The level of insight participants had towards the role of the nurse may cause some concern as to what the participants thought they were applying for, when applying for the programme. Equally, this is interesting as to what the public perception of the role of the nurse is; specifically linking to what draws people to the profession. Lucy reiterates this,

even after working as a senior healthcare assistant (HCA) on one ward for 10 years, I don’t think I had any idea of the bigger picture, I suppose I just saw the nurses as people who were always busy and spoke a lot to the doctors, I just didn’t realise that different settings have different demands, and the amount of pressure and responsibility that you have as a nurse . . . I don’t know if I had the insight would I actually want to do this as it’s just so much responsibility. (Lucy)

Overall, the findings suggest that participants, regardless of their healthcare experience, demonstrate they had little appreciation of the role of the nurse when commencing the programme. This finding is interesting considering the programme consists of 50% practice and 50% theory, with the year 1 modules and the preparation for practice placements including a lot of preparatory work for students before they move into clinical practice. Yet, there may well need to be curriculum development in response to this to ensure that students starting the programme in the first year understand the important role of the nurse and how much responsibility there is.

### Nursing socialisation

Participants suggested that they underwent a period of socialisation, in terms of working with the staff in the clinical areas, and by this they meant the period of time to allow for orientation on the clinical areas. For some, this was as little as a few days, and others required a longer period. Yet it was very clear that while the students may be on duty with their assessors to achieve maximum time to be assessed and learn from them, they work more closely with the healthcare assistant (HCA)’s and when on duty they utilise the RNs as a point of information if they have any queries. It appears that they used this as a way of understanding what the role of the nurse was. Interestingly participants did not discuss the other members of the healthcare team who they learn from. Nine participants demonstrated who they learned from, Nicola highlights her thoughts, ‘The good thing about going onto so many different placements is that I feel that we benefit from getting involved with all of the different staff and learn how they work . . . we work so much with the HCA’s’ (Nicola). Hayley discusses how she not only learns how to be a nurse from working with nursing staff, but that she also learns other skills such as how to work in a team. Teamwork was discussed and participants highlight that this is something that is not taught within university but is learned while working within clinical teams in practice. Hayley, who had previous clinical experience, discusses this,

you quickly learn from all of these placements how to become part of the team, being part of the nursing team is a massive deal and can make you learn very quickly about how to be a nurse . . . this is the type of thing that you don’t get in university . . . they can teach you all the knowledge but being a nurse comes from working with nurses. (Hayley)

This demonstrates the approach to learning and working in practice that participants use, to understand what the role of the nurse is and to gain the professional socialisation that comes from working in practice. Barbara states, ‘I have found the best thing to do is to find yourself a good new staff nurse or a good nursing assistant and they show you the ropes and after a while you’re fine’ (Barbara). Equally, Susan, a final year student, highlights a valuable finding which many other participants discussed, around their lack of insight into the depth of knowledge required to be a nurse, ‘A big part of being a nurse is not actually about knowing . . . you really don’t need to know a lot just more about how the ward works and then you’re fine’ (Susan).

Findings around socialisation suggest that it is not just learning the process and skills from assessors to be able to do the job of the nurse but also about how to behave and portray the role of the nurse, which is important to participants within their education.

Amy demonstrates this,

I think the more you work with nursing staff the better because it’s from them that you learn to act, maybe it’s about acting and portraying yourself as a nurse that helps me to feel ready and have more confidence, rather than the stuff I learn at university. (Amy)

Learned behaviours are significant in this period of socialisation and participants appear to learn the role of the nurse from practice colleagues. Furthermore, this appears to be beneficial to participants, in view of developing confidence. There was a lack of understanding of the role of the nurse at the beginning of the programme, and this does continue for some participants as they move towards their registrant status.

### View of the role of the nurse for now and the future

Seven participants describe what they perceive the role of the nurse as now, and what they believe the role of the nurse to be in the future and what the public sees as a nurse’s role. Susan demonstrates how she perceives that the role of the nurse is misunderstood by the public and this may be a challenge for nurses’ identity and public perception when recruiting future nurses, ‘I just don’t think that people know what nurses do, and I actually think that this is a challenge within modern working’ (Susan).

Maureen highlights that she believes the role of the nurse has evolved from what it was in the past, and that the profession is challenging in terms of accountability and responsibility, although she suggests this has not been realised by the public, ‘I don’t think the role of the nurse, from what I can see, is ever going to be really like it was 50 years ago, we have so much responsibility that the general public don’t realise’ (Maureen). Lisa suggests that due to the ever-changing role of the nurse, nurses find it difficult to fully understand their role, as there are often additional aspects added within their remit. Likewise, many tasks and skills that are currently perceived as advanced, were not undertaken by nurses or were only ever undertaken by nurses in specialist roles.

Participants also describe nurses as ‘cheap labour’. This is in relation to the frustration of the student role and in terms of their future role as a registrant. This may be a reference to the advancement of the nursing roles, and their perception that some nurse’s roles are in place of doctors. More importantly this is a potential challenging issue as the students may not understand the scope of practice as a nurse of it is not embraced. Lisa highlights this,

We do lots of the doctor’s jobs I think this will be the case more than ever actually, I should have just trained to be a doctor at least then I would know what my scope of practice was and would be paid for it, it’s sometimes like we’re just very very cheap labour. (Lisa)

While the findings are significant in terms of what student nurses perceive the role of the nurse to be, Phil also highlights that nurses have multiple roles and predicts it will change in the future, ‘Our job roles are so diverse, and I am not sure how the roles are going to develop further within the next 20 years or so, and this worries me’ (Phil).

This in terms of students having an awareness of the wider nursing professional landscape, and how nurses fit into the future healthcare system. As such, this would mean that the curriculum should be flexible and responsive. However, many participants did talk considerably about nurses undertaking tasks and there was little talk about the delivery of holistic care. Participants demonstrated they had little understanding of the role of the nurse when commencing the programme, and this was no different between participants who did, and did not, have healthcare experience. To assist with understanding what the role of the nurse is, participants undergo a period of socialisation, through this, participants also used this as a time to learn the role of the nurse. Participants then demonstrated that they have a reasonable understanding of the role of the nurse at the point of qualification, and that they understand the potential future nurses’ role.

## Discussion

The findings of this research demonstrate how, when commencing the nursing programme, many participants had a limited understanding of what the role of the nurse was. Despite this, participants applied for the programme and were successful in attaining their place. This was apparent even with participants who had healthcare experience. Even at the point of registration, some participants still demonstrate how they have a restricted view of the role of the nurse. This is apparent by the nature of participants’ responses within the findings, who discuss nursing and care simply as undertaking tasks, and the notion of holistic nursing is discussed minimally. Simply undertaking tasks appears to be what many participants believe the role of the nurse to be. It is concerning that this is the perception of what the role of the nurse is, and while there may be an element of realism in terms of the need to undertake tasks, to conduct investigations and to provide treatments for patients, it could be argued that this is not simply the role of the nurse. As the literature review discusses (DH, 2015; ICN, 2016; NMC, 2018; Smith, 1992; Willis, 2015), nursing is so much more than simply completing tasks and is fundamentally about providing holistic care to patients and their families.

Caring for patients holistically is not central to the modern nurses’ role, from the participants’ perspective. Looking to the future, participants do discuss how they feel the role of the nurse will change, and nurses will have a different role, which leaves participants unsettled. Perceptions by participants regarding the future role of the nurse refers to undertaking skills, rather than providing the holistic care. While nursing as a role has changed to mean that nurses undertake more skills and tasks which were once considered advanced skills, it is highly concerning that the holistic nature of nursing as part of the role has been lost. This necessity to simply undertake tasks may have derived from staffing shortages, the complexities of patients’ illnesses, coinciding with the need to deliver rapid care to patients and driven by the demands of the current healthcare system (NHS England, 2013). Previous conceptions of the nurses’ role may have come from ideas formulated from past idylls. It is not to say that nursing as a profession should not be advancing, as the healthcare system requires, but that the central premise of being a nurse, based upon the perceptions of this group, is that nurses are members of the healthcare system who simply undertake clinical tasks, rather than providing care. Part of a student’s understanding of what nurses do comes from assessors and the socialisation from learning in clinical practice. The student nurses in this study perceive that acting in the role of the nurse is essential. Participants have a limited understanding of what the role of the nurse is. Half of the time students spend on their programme is within clinical practice, and it is here that professional learning occurs, which is where they formulate their understanding of what the role of the nurse is. However, it should not be discounted that learning from nurses and role modelling also should happen while students are at university. This is based upon working with their assessors and observing other RNs’ practice. The RNs who act as assessors to student nurses, and who assess student performance, are pivotal in allowing students to understand the nursing role.

Part of the preparation that student nurses undertake to practice is the professional socialisation within clinical areas that occurs during the practice time. This implicit aspect of the curriculum exposes students to the skills and knowledge which cannot be taught, such as behaviours and role modelling. Cohen (1981) discussed how professional socialisation is important as this is the part of the curriculum where the student will both gain the professional knowledge and skills and form their professional identity. Cohen (1981) does not identify for student nurses transitioning to registrant, it is the day-to-day workings of how clinical areas function that are perceived by students as important to allow them to succeed in their role. This research has found that this is very important to student nurses, in terms of their pre-registration education. This is significant in terms of students understanding what the role of the nurse is, which is not about simply undertaking tasks, but about providing holistic care to patients. Their perception of nursing simply being a task orientated role is concerning, because nursing requires more skill, knowledge and finesse than simply undertaking tasks. It appears that these attitudes are being perpetuated by the socialisation that students undergo, but it was unclear from the interviews where these behaviours arose from, that is, the RNs, the nursing assistants or the wider multidisciplinary team. The participants did note that they spent a significant amount of their early programme time with nursing assistants. Eraut (1994) explains how there are several methods of preparatory training by professions, such as nursing, which lead to academic qualification following study at university and learning from experts in practice. The mix of studying theory at university and learning in practice is intended to allow students to register with a good level of competence. The NMC (2010) believe that this model ensures student nurses would then leave university following education as a professional nurse with an adequate standard of clinical competence. However, what may not have been noted previously is the significance of the socialisation that student nurses undergo when in the practice arena. This is to allow students to build their knowledge and skills and develop the tacit knowledge learned through practice time. Working within clinical practice consists of not only gaining professional knowledge but also understanding the cultural practices of each clinical area. It was clear from this research that student nurses spend a lot of time learning from nursing assistants while in clinical practice, learning essential skills and understanding how clinical areas work. Student nurses gain much experience from these members of the wider team, in terms of clinical cultural practices and the delivery of fundamental care. The acquisition of knowledge and delivery of fundamental care, which has come from nursing assistants, was an unexpected and significant finding in terms of the education of student nurses. Willis (2015) acknowledges that unqualified nursing assistants with little access to formal training provide 60% of hands-on care to patients, yet it is largely un-acknowledged that student nurses learn so much from these unqualified staff. Student nurses should be gaining the underpinning knowledge and skills to practice effectively from RNs work most of their time with nursing assistants, may contribute to student nurses perceive nursing to be a task orientated role. Eraut (1994) discussed how the period in practice contributes to a person’s knowledge base and socialisation to the occupation. The significance of this may not have been fully recognised in nursing, in terms of who within the healthcare team it is that student nurses learn from.

### Limitations

The limitations of this study are common with any other study that uses a qualitative approach (Qu and Dumay, 2011). Limitations of this research relate to this being a sample of one field of nursing from one HEI. However, this smaller in-depth study does allow for an in-depth illumination of the phenomenon of what this group of student’s perceptions are. An acknowledgement has been made that a sample size of small numbers has been used, yet there has been a rationale for why this was the case. Furthermore, it has also been acknowledged that other nursing education research (Watts and Davies, 2014) has utilised smaller numbers, which provides a justification that this is an acceptable approach.

### Recommendations

A recommendation would be to ensure that student nurses work most of their practice time with an RN assessor.

Further work needs to be undertaken around informing candidates at recruitment events what the role of the nurse is and to allow candidates who have a good level of understanding and make the correct decision when applying for university places, recruitment process to ensure that current potential candidates are being recruited who understand the role of the nurse, in addition to this potential candidate may be lost. Additionally, semester one could be used to reinforce what nurses do in their role and how delivering care and conducting tasks which relate to patient care is the main goal of a nurse and the healthcare team. In addition to this, the nursing curriculum should have a heavy focus upon the holistic role of the nurse.

## Conclusion

The findings have been discussed in relation to the extent of which final year student nurses perceive their perception of the role of the nurse to have changed over the course of their programme. However, they still do not demonstrate that they fully perceive the role of the nurse to be a holistic caregiver, instead they perceive the nurse as somebody who completes tasks. It is perceived that nurses undertake a very task orientated role, and holistic care was mentioned by the participants minimally.

Implications of this potentially mean that the role of the nurse may be forever changed if this perception continues with each new generation of nurses. It was clear through the research that students spend a significant amount of time working with HCAs in clinical practice. It appears that particularly in the early parts of the pre-registration programme, students are informally assessed by HCAs, and potential implications of this may be that students are missing out on learning opportunities from RNs. A further significant contribution to knowledge is the level of professional learning and socialisation that takes place, particularly in terms of where and who the learning occurs from.

It is clear from this research that student nurses when reflecting upon their experience of the programme do report changes and developments in their perceptions of the nurses’ role over the course of their degree. This is because the students have had experiences of being socialised into the role of the nurse through clinical practice, in turn, this has allowed developing confidence and assertiveness in their practice. However, while students do perceive that their understanding of the role of the nurse has changed through their progression and that they are ready to qualify, participants’ perceptions of the role of the nurse may be misaligned to that of the public. Participants perceive nurses to be task orientated rather than holistically focused. This assumption may be because of the amount of time that students spend with HCAs while out on placement. In view of this, there are considerable implications that this may have on the nursing profession and on patient care, should every student nurse share the same assumption when they then move onto the professional register.

**Key points for policy, practice and/or research**

- The 18 adult field pre-registration nursing students who participated in this study perceive the role of the nurse to be task orientated rather than holistic, and as such this may well lead to a tension for their perception of what the role of the nurse is within the nursing profession when they move forward.
- Student nurses learn a lot from unregistered healthcare professionals, and this may cause a challenge when they move into a RN role.
- Internationally nursing bodies should consider the professional image that they portray nurses as and reflect upon what roles nurses are doing.
- Holistic nursing care within its truest sense may be diminished to provide advanced nursing roles. With the advancement of nursing roles through advanced practice, this may lead to ‘care’ as a nursing role being lost and is something that the nursing professional needs to protect for its identity.
